# The Interaction Between Mitophagy Dysregulation and the Diabetic Bladder Microenvironment

**DOI:** 10.1111/1753-0407.70199

**Published:** 2026-03-06

**Authors:** Shi Li, Zongyao Fan, Zheng Duan, Jun Xue, Zhongqing Wei

**Affiliations:** ^1^ Department of Urology The Second Affiliated Hospital of Nanjing Medical University Nanjing China; ^2^ Department of Urology The Second Clinical Medical College of Nanjing Medical University Nanjing China

**Keywords:** bladder microenvironment, DBD, mitophagy dysregulation, oxidative stress, PINK1/Parkin pathway

## Abstract

Diabetic bladder dysfunction (DBD) is a prevalent and multifactorial urological complication of diabetes, with pathogenesis driven by complex interactions between hyperglycemia‐induced oxidative stress, mitochondrial dysfunction, and bladder microenvironment dysregulation. Mitophagy, a selective autophagic process critical for mitochondrial quality control, has been linked to various metabolic diseases, but its precise role and the bidirectional interactions with the diabetic bladder microenvironment remain underexplored. This review outlines a novel, self‐reinforcing feedback loop central to DBD progression. In this cycle, hyperglycemia impairs both the PINK1/Parkin‐mediated mitophagy pathway and ubiquitin‐independent pathways like FUNDC1 under hypoxic conditions, leading to the accumulation of damaged mitochondria. Mitochondrial dysfunction then exacerbates microenvironmental damage through excessive mitochondrial reactive oxygen species (mtROS) production, release of damage‐associated molecular patterns (DAMPs), and activation of the NLRP3 inflammasome, which further drives inflammation, fibrosis, and extracellular matrix (ECM) remodeling. This aggravated microenvironment inhibits mitophagy, thereby accelerating the pathogenic cycle. Beyond elucidating this loop, this review suggests that targeting it offers a promising therapeutic strategy. A breakthrough in DBD treatment may necessitate a combined approach that both restores mitophagy and modulates the microenvironment. Additionally, this study critically reviews several promising, yet underexplored, interventions, including pharmacological mitophagy activation with urolithin A, NACHT, LRR, and PYD domains‐containing protein 3 (NLRP3) inflammasome inhibition via MCC950, and advanced techniques like nanoparticle‐mediated PINK1 mRNA delivery and CRISPR/Cas9‐based Parkin gene editing. Future research should incorporate spatial transcriptomics to resolve cellular heterogeneity, develop targeted nanodelivery systems, and establish mechanism‐driven, highly specific combination therapies to enable precision medicine for DBD.

## Highlights

- A novel bidirectional feedback loop connects mitophagy dysregulation with the deterioration of the diabetic bladder microenvironment.
- Hyperglycemia inhibits PINK1/Parkin‐mediated mitophagy, leading to ROS accumulation and fibrosis.
- Therapeutic synergy is achieved by simultaneously targeting both mitophagy and microenvironment modulation.
- Diabetic microenvironmental factors (hypoxia/AGEs/inflammation) impair mitophagy via FUNDC1 and lysosomal dysfunction.
- Spatial transcriptomics and nanodelivery systems are essential for advancing precision therapies in DBD.

## Graphical abstract

Hyperglycemia suppresses the PINK1/Parkin pathway, impairing mitophagy and triggering ROS accumulation. This activates NLRP3 inflammasome and AGEs‐RAGE axis, driving bladder inflammation and fibrosis. A bidirectional positive feedback loop forms between mitophagy dysregulation and microenvironment deterioration, accelerating diabetic bladder dysfunction.

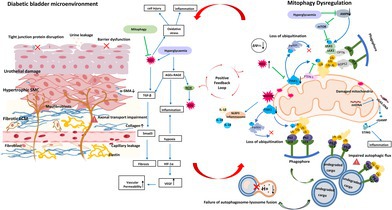

## 1 Introduction

### 1.1 Overview of Diabetic Bladder Dysfunction (DBD)

DBD is a prevalent urological complication in diabetes, marked by distinctive pathological features and clinical progression, which can be divided into two stages. In the early phase, the detrusor muscle becomes hyperreactive, while, as the disease advances, bladder function deteriorates, resulting in reduced bladder contractility. These dynamic changes lead to characteristic urodynamic abnormalities of DBD. Initially, symptoms primarily manifest during the storage phase, including frequent urination and urgency incontinence, both day and night. In later stages, the disease progresses to voiding‐phase dysfunction, characterized by increased postvoid residual urine and diminished bladder emptying efficiency [1]. Epidemiological studies indicate that the risk of DBD increases significantly in patients with prolonged diabetes, with its severity correlating positively with disease duration [2]. The pathophysiology of DBD involves chronic hyperglycemia, which mediates tissue damage through several pathological pathways. Thus, diabetic patients with poor blood sugar control are at a higher risk for DBD. Research highlights the abnormal activation of the polyol pathway, disruption of the protein kinase C (PKC) signaling pathway, and excessive accumulation of advanced glycation end products (AGEs) as key pathogenic factors. These mechanisms induce oxidative stress responses in bladder smooth muscle cells (BSMCs), urothelial cells in the mucosal layer, and bladder afferent nerve terminals, leading to organ dysfunction [1, 3].

DBD significantly impacts patients' quality of life. The loss of bladder filling sensation in diabetic patients is closely associated with urothelial barrier disruption. A marked reduction in tight junction protein expression between urothelial cells disrupts neural signal transmission, impairing the micturition reflex [4]. Diabetic peripheral neuropathy also affects bladder afferent nerves, inhibiting axonal transport and reducing nerve efficiency. This results in impaired bladder sensation and urinary dysfunction [5]. These pathological changes not only affect the bladder's mechanical properties but may also lead to bladder wall remodeling, altering its compliance [6].

### 1.2 Introduction to Mitophagy

Mitophagy, a highly conserved intracellular homeostasis mechanism, targets and degrades dysfunctional mitochondria to maintain mitochondrial quality control. The molecular regulatory pathway involves several key steps. Damaging factors, such as mitochondrial membrane potential loss or ROS accumulation, trigger intracellular damage recognition signals. These signals activate the formation of autophagosomes, which ultimately facilitate the degradation of dysfunctional mitochondria via the lysosomal system, thereby renewing organelles within the cell [7, 8]. In mammalian cells, mitophagy regulation primarily occurs through two pathways: the ubiquitin‐dependent pathway and the ubiquitin‐independent pathway. The ubiquitin‐dependent pathway involves the PINK1/Parkin system. Mitochondrial outer membrane depolarization results in the accumulation of PTEN‐induced kinase 1 (PINK1) on the outer mitochondrial membrane, which recruits and activates Parkin. Parkin activation triggers the ubiquitination of mitochondrial surface proteins, leading to the formation of autophagosomes, mediated by autophagy receptors [9, 10].

Recent research highlights the critical role of mitophagy in metabolic diseases such as diabetes and obesity. By maintaining mitochondrial network homeostasis and balancing energy metabolism, mitophagy has emerged as a promising therapeutic target. Hyperglycemia impairs mitophagy, resulting in the accumulation of dysfunctional mitochondria, which exacerbates ROS production and disrupts cellular energy metabolism [11]. For example, in diabetic nephropathy models, downregulation of PINK1 expression significantly reduces mitophagy efficiency, leading to increased apoptosis of renal tubular epithelial cells [12]. Moreover, mitophagy plays a role in diabetes‐related inflammatory responses by regulating immune cell functions, highlighting its multifaceted involvement in pathological processes [8]. In 2016, Professor Yoshinori Ohsumi was awarded the Nobel Prize in Physiology or Medicine for his groundbreaking research on autophagy. His discovery of the molecular mechanisms underlying autophagy has laid the foundation for translational research into mitophagy‐based therapeutic strategies [13].

### 1.3 Bladder Microenvironment

The bladder microenvironment consists of several key components, including the urothelium, smooth muscle, neurovascular network, and extracellular matrix (ECM), whose interactions are essential for both normal bladder function and the development of pathological changes.

The urothelium primarily serves as a mechanical barrier but also plays a critical role in bladder function regulation by mediating bladder filling perception and neural regulation. This is achieved through the expression of transient receptor potential channels and purinergic receptors [14]. Smooth muscle significantly impacts bladder function, as its contraction and relaxation directly affect bladder performance during both storage and voiding phases. Alterations in the ECM, a critical component of the bladder microenvironment, are closely associated with the onset and progression of various bladder diseases. Research suggests that increased ECM stiffness can promote BSMC proliferation through modulation of the YAP/Smad3 signaling pathway, thereby exacerbating bladder dysfunction during fibrosis [15]. Additionally, the neurovascular network supports the structural integrity and function of the bladder microenvironment. It is involved in regulating normal bladder function and plays a key role in the repair process following bladder injury [16]. Thus, the various components of the bladder microenvironment interact in a complex manner to maintain normal bladder function. In pathological conditions, these components contribute to the progression of bladder diseases through diverse mechanisms. A schematic representation of the structural organization and molecular composition of the normal bladder microenvironment is provided in Figure 2.

### 1.4 Rationale for the Review

Although the clinical progression of DBD is well‐established, a comprehensive analysis of the molecular signaling pathways, particularly those related to organelle quality control and microenvironmental interactions, remains insufficient. Research has predominantly focused on direct hyperglycemic damage, such as oxidative stress and neuropathy. However, more intricate intracellular mechanisms, especially the imbalance in mitophagy and its bidirectional regulatory network with the bladder microenvironment, remain poorly understood. This review seeks to address this gap by synthesizing evidence on these complex interactions and proposing a novel theoretical framework for precision therapeutics in DBD. Recent studies have shown that defects in mitochondrial autophagy worsen microenvironmental disruption through ECM remodeling, while hypoxia and inflammatory responses establish a pathological feedback loop by inhibiting the PINK1/Parkin pathway [17, 18]. Although mitophagy‐targeting strategies have demonstrated cross‐organ protective effects in diabetic complications, their specific application to DBD warrants further investigation [19, 20]. This review integrates multidisciplinary evidence to explore the molecular network between mitophagy and the microenvironment, offering a theoretical foundation for precision diagnostics and treatment strategies for DBD.

This review goes beyond summarizing isolated pathways to define a previously unrecognized bidirectional feedback loop between mitophagy dysregulation and microenvironmental deterioration. Unraveling this self‐perpetuating cycle represents a significant conceptual advancement and identifies multiple novel therapeutic targets, which are explored in detail herein.

### 1.5 Objective of the Review

The review synthesizes current evidence into a new pathogenic framework for DBD, focusing on the dynamic, bidirectional interaction between mitophagy dysregulation and microenvironmental decline. The primary objective is to elucidate the core components of this feedback loop, beginning with how hyperglycemia suppresses canonical pathways such as PINK1/Parkin and disrupts non‐canonical pathways like FUNDC1. Further analysis will clarify how the resulting mitochondrial dysfunction, marked by excessive mtROS and mtDNA release, promotes NLRP3 inflammasome activation and pro‐fibrotic signaling, thus exacerbating microenvironmental damage. Ultimately, the review critically evaluates the therapeutic potential of disrupting this vicious cycle by targeting underexplored areas, including specific mitophagy receptors, lysosomal function, and the NLRP3 axis, and by assessing innovative strategies ranging from combinatorial pharmacology to advanced nanodelivery and gene editing approaches.

### 1.6 Literature Search Strategy

A comprehensive literature search was conducted using electronic databases (PubMed/MEDLINE, Web of Science, Google Scholar) for articles published up to October 2025. Search terms included various combinations of “mitophagy,” “autophagy,” “diabetic bladder dysfunction,” “diabetic cystopathy,” “bladder microenvironment,” “oxidative stress,” “fibrosis,” “PINK1,” “Parkin,” “FUNDC1,” “NLRP3 inflammasome,” and “diabetes complications.” The search was limited to English‐language original research articles and reviews. Additionally, the reference lists of retrieved articles were manually reviewed to identify further relevant studies. Emphasis was placed on studies that elucidated molecular mechanisms, utilized in vivo or in vitro DBD models, and investigated therapeutic interventions targeting mitophagy or the microenvironment. This strategy ensured a thorough and up‐to‐date synthesis of current knowledge on the subject.

## 2 DBD: From Clinical Presentation to Pathological Remodeling of the Bladder Microenvironment

### 2.1 Clinical Trajectory of DBD: From Overactivity to Underactivity

DBD manifests a biphasic clinical progression that mirrors underlying pathological shifts within the bladder wall. In the early phase, patients primarily experience storage symptoms, including urinary frequency, nocturia, and urgency incontinence, indicative of detrusor overactivity and heightened afferent sensitivity [1, 21]. As DBD advances, a shift toward voiding dysfunction occurs, characterized by poor emptying efficiency, increased post‐void residual urine, and a weakened urine stream, reflecting detrusor decompensation and reduced contractility [1, 22]. This evolution from an overactive to an underactive phenotype is underpinned by profound alterations across all components of the bladder microenvironment, as detailed below.

### 2.2 Hyperglycemia‐Driven Pathological Remodeling of the Bladder Microenvironment

#### 2.2.1 Urothelial Dysfunction and Barrier Breakdown

The urothelium, a critical barrier and sensory interface, is a prime target in DBD. Hyperglycemia compromises the physical barrier function through downregulation of tight junction proteins, increasing permeability to urinary toxins and initiating inflammatory cascades [23, 24]. Concurrently, diabetic insult disrupts urothelial signaling pathways integral to bladder sensation. This includes dysregulation of transient receptor potential and purinergic receptors, impairing the normal afferent signaling necessary for the micturition reflex [3, 14, 25]. Furthermore, impaired insulin receptor signaling within the urothelium weakens its innate antimicrobial defenses, contributing to the heightened susceptibility to urinary tract infections observed in diabetic patients [26, 27]. These collective deficits—barrier loss, sensory dysfunction, and immune compromise—directly underlie the storage symptoms and sensory abnormalities characteristic of early DBD.

#### 2.2.2 Smooth Muscle Phenotype Switching and Contractile Impairment

BSMCs undergo detrimental phenotypic switching from a contractile to a synthetic state under diabetic conditions, a key driver of voiding dysfunction. Hyperglycemia and the resultant microenvironment inhibit insulin receptor signaling in SMCs, leading to aberrant proliferation and significantly reduced contractile force, which manifests as decreased voiding efficiency [28]. This phenotype switch is orchestrated by pro‐fibrotic pathways, most notably TGF‐β/Smad3, which represses contractile markers like α‐smooth muscle actin (α‐SMA) while upregulating the synthesis of ECM components such as collagen I and III [29]. Additionally, increased ECM stiffness itself acts as a pathological stimulus, activating the YAP/Smad3 axis to further promote SMC proliferation and fibrogenic activity, thereby creating a feedforward loop that exacerbates tissue fibrosis and bladder wall stiffening [15]. Functional alterations in calcium channels, potentially influenced by this altered milieu, may also contribute to detrusor instability and contractile failure [30].

#### 2.2.3 Neurovascular Damage and Altered Sensation

Diabetic neuropathy and microangiopathy critically disrupt the neural and vascular networks essential for normal bladder function. Afferent nerve damage, driven by oxidative stress and axonal transport deficits, blunts the perception of bladder filling—a defect reflected in elevated urodynamic sensation thresholds [1, 31, 32]. Efferent autonomic neuropathy contributes to insufficient detrusor contraction during voiding [23]. Concurrently, microvascular endothelial dysfunction induces chronic hypoxia within the bladder wall. This hypoxic stress stabilizes hypoxia‐inducible factor‐1α (HIF‐1α), which upregulates vascular endothelial growth factor (VEGF). While intended to promote angiogenesis, this response often results in leaky, dysfunctional vessels, exacerbating tissue edema and hypoxia, and further compromising the delivery of oxygen and nutrients [33, 34]. This neurovascular insufficiency synergistically impairs both the sensory signaling and the energetic supply required for effective detrusor contraction.

#### 2.2.4 ECM Remodeling and Fibrosis

A hallmark of advanced DBD is pathological ECM remodeling, which directly compromises bladder compliance and contractility. Diabetic stimuli disrupt the balance between collagen synthesis and degradation, leading to excessive deposition of fibrillar collagen I and III and disorganization of elastic fibers [35]. This fibrosis increases tissue stiffness, which mechanically impedes bladder expansion during filling, contributing to reduced compliance and elevated storage pressures [36]. The stiffened ECM is not merely a passive outcome but an active driver of pathology; it engages integrin‐mediated signaling to promote further fibroblast‐to‐myofibroblast differentiation and SMC proliferation, thereby amplifying fibrosis [37]. Key signaling pathways such as NF‐κB‐JMJD3 are activated in this context, fueling a pro‐fibrotic transcriptional program that sustains collagen accumulation and smooth muscle hyperplasia [38]. Ultimately, this self‐perpetuating fibrotic process leads to a non‐compliant, poorly contracting bladder, characteristic of the decompensated stage of DBD.

#### 2.2.5 Key Molecular Drivers: Oxidative Stress, AGEs, and Inflammation

The pathological remodeling described above is propelled by a self‐reinforcing triad of molecular pathologies entrenched in the hyperglycemic milieu. Foremost, mitochondrial dysfunction drives a state of persistent oxidative stress, characterized by the overproduction of ROS, particularly mtROS. These mtROS inflict direct damage on cellular components while simultaneously acting as potent signaling molecules that disrupt normal bladder function [1, 39]. This oxidative environment synergizes with the accumulation of AGEs, which form under sustained hyperglycemia. AGEs contribute to tissue stiffening through protein cross‐linking and, upon binding to their receptor, activate downstream pathways that further fuel oxidative stress and inflammatory responses [40, 41]. Critically, both oxidative stress and AGEs converge to sustain a state of chronic, low‐grade inflammation. Key inflammatory mediators such as TNF‐α and IL‐1β are elevated [42]. Notably, mtROS and the consequent release of mtDNA serve as potent DAMPs that activate the NLRP3 inflammasome. This activation leads to the caspase‐1‐dependent maturation of IL‐1β and IL‐18, cytokines that directly promote fibrotic pathways and tissue injury [42, 43]. Thus, oxidative stress, AGEs, and inflammation do not operate in isolation; they intertwine to form a vicious, self‐amplifying network that constitutes the core molecular engine of microenvironmental deterioration in DBD.

## 3 Mitophagy: Mechanisms and Its Role in Cellular Homeostasis

### 3.1 Molecular Mechanisms of Mitophagy

Mitophagy, a selective autophagic process essential for mitochondrial quality control, is primarily governed by two distinct classes of molecular pathways: ubiquitin‐dependent and ubiquitin‐independent mechanisms [7].

The ubiquitin‐dependent pathway is predominantly orchestrated by the PINK1/Parkin axis. Under conditions of mitochondrial damage, particularly inner membrane depolarization, the serine/threonine kinase PINK1 is stabilized on the outer mitochondrial membrane. This accumulation enables PINK1 to phosphorylate both ubiquitin molecules and the cytosolic E3 ubiquitin ligase Parkin. Phosphorylation serves as a critical activation signal, recruiting Parkin to the damaged mitochondrion and potently activating its ubiquitin ligase activity [9, 44, 45].

Once activated, Parkin ubiquitinates numerous proteins on the mitochondrial outer membrane. These polyubiquitin chains then function as a potent recognition signal for selective autophagy. This signal is specifically decoded by autophagy adaptor proteins such as p62/SQSTM1, OPTN, and NDP52. These adaptors bridge the ubiquitin‐tagged mitochondrion to the lipidated form of LC3 known as LC3‐II, which is embedded in the expanding phagophore membrane. This molecular bridging event facilitates the engulfment of the organelle into a forming autophagosome [10, 46].

In parallel, ubiquitin‐independent or receptor‐mediated pathways offer an alternative route for mitochondrial clearance. This strategy employs a suite of resident outer mitochondrial membrane proteins that function as dedicated mitophagy receptors, including FUNDC1, BNIP3, and NIX. Each of these receptors harbors a conserved LC3‐interacting region. Upon activation by specific cellular stresses, this exposed region engages directly with LC3 on the phagophore, tethering the mitochondrion to the autophagy machinery without the requirement for prior ubiquitination [47, 48].

**FIGURE 1:**
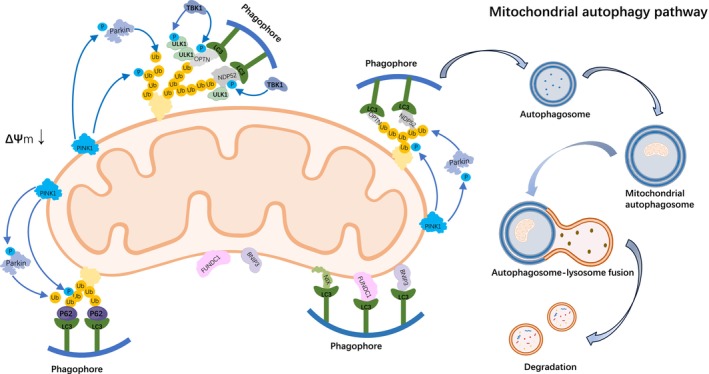
Molecular mechanisms of mitophagy in diabetic bladder microenvironment. This figure illustrates the core molecular pathways that regulate mitophagy, a selective autophagic process essential for mitochondrial quality control. Dysfunctional mitochondria are targeted for degradation by damage signals, such as membrane depolarization or oxidative stress, through two primary mechanisms. The ubiquitin‐dependent pathway, shown on the left, is orchestrated by PINK1 and Parkin. PINK1 accumulates on the depolarized mitochondrial outer membrane and recruits Parkin, which ubiquitinates outer membrane proteins. These ubiquitin tags are recognized by autophagy adaptor proteins such as p62, OPTN, and NDP52, which link the damaged mitochondria to LC3‐positive phagophores, facilitating mitophagosome formation. Simultaneously, the ubiquitin‐independent pathway, shown on the right, is mediated by outer membrane receptors like FUNDC1, BNIP3, and NIX. These receptors contain an LC3‐interacting region that directly binds LC3 on the phagophore, promoting mitochondrial engulfment. This pathway is particularly activated under hypoxic conditions. The mature mitophagosome then fuses with a lysosome, where the encapsulated mitochondrial components are degraded, completing the organelle turnover process and maintaining cellular metabolic homeostasis. ΔΨm, mitochondrial membrane potential; BNIP3, BCL2/adenovirus E1B 19 kDa protein‐interacting protein 3; FUNDC1, FUN14 domain‐containing protein 1; LC3, microtubule‐associated protein 1 light chain 3; NDP52, nuclear dot protein 52; NIX/BNIP3L, BNIP3‐like protein; OPTN, optineurin; p62/SQSTM1, sequestosome 1; PINK1, PTEN‐induced kinase 1; TBK1, TANK‐binding kinase 1; Ub, ubiquitin; ULK1, Unc‐51‐like autophagy‐activating kinase 1.

The activation of these receptors is highly context‐dependent. For instance, FUNDC1 is notably upregulated and activated under hypoxic conditions, representing a key adaptive response to low oxygen [49, 50]. The precise regulatory interplay and relative contribution of these distinct pathways—the ubiquitin‐dependent PINK1/Parkin system and various receptor‐mediated routes—within the complex pathological landscape of diseases such as diabetes remain an active and critical area of investigation. A summary of these core molecular pathways, including both the PINK1/Parkin‐dependent and receptor‐mediated ubiquitin‐independent mechanisms, is illustrated in Figure 1.

### 3.2 The Role of Mitophagy in Cellular Health

Mitophagy plays an essential role in regulating energy metabolism, responding to oxidative stress, and preventing apoptosis [18]. In energy metabolism, mitophagy helps optimize mitochondrial network function by removing damaged components, thereby supporting efficient ATP production [51, 52]. This process works in concert with mitochondrial dynamics, such as fusion and fission, to maintain a healthy organelle pool [18].

Regarding oxidative stress, mitophagy lowers cellular ROS levels by clearing mitochondria that have become excessive ROS producers, protecting the cell from oxidative damage [53]. Furthermore, mitophagy is closely tied to anti‐apoptotic regulation. By eliminating mitochondria that could release cytochrome c, it inhibits the mitochondrial‐dependent apoptosis pathway and promotes cell survival [54, 55]. Thus, mitophagy is a critical guardian of cellular homeostasis.

## 4 The Interaction Between Mitophagy Dysregulation and the DBD Microenvironment

### 4.1 The Impact of the Microenvironment on Mitophagy Dysregulation

Mitophagy dysregulation is a common pathogenic feature across diabetic complications, such as nephropathy and neuropathy, where it amplifies cellular damage [12, 56]. In DBD, this dysregulation engages in a unique, bidirectional crosstalk with the local tissue microenvironment. Specifically, the diabetic bladder microenvironment—characterized by oxidative stress, hypoxia, and inflammation—actively suppresses mitophagy. This suppression occurs through the impairment of key pathways, including the inhibition of the PINK1/Parkin axis and the disruption of ubiquitin‐independent mechanisms such as FUNDC1, alongside compromised lysosomal function [57, 58, 59].

Oxidative stress, chronic inflammation, and hypoxic conditions in the diabetic bladder microenvironment significantly impair mitophagy, creating a complex regulatory network. Hyperglycemia activates the polyol pathway and the AGEs‐RAGE axis, leading to excessive ROS production, which directly damages mitochondrial membrane integrity and inhibits the PINK1/Parkin pathway [60]. The accumulation of AGEs in the diabetic environment causes mitochondrial membrane depolarization, which impedes the ubiquitination of mitochondrial proteins—a critical step for initiating mitophagy [58]. The absence of ubiquitination markers prevents the recognition and clearance of damaged mitochondria, leading to failure in mitochondrial quality control within the cell [61]. Moreover, ROS can suppress autophagy‐related genes, triggering defects in autophagic function [62]. This inhibition may be mediated through several signaling pathways, including the suppression of the AMPK‐mTOR pathway, which affects autophagic activity [57].

Hypoxia is a hallmark of the diabetic microenvironment and regulates the ubiquitin‐independent mitophagy pathway, such as FUNDC1, through the HIF‐1α pathway [49]. Under hypoxic conditions, the LIR domain of FUNDC1 binds to LC3, enhancing the selective clearance of damaged mitochondria [50]. Chronic hyperglycemia induces both hypoxia and oxidative stress, which disrupt mitophagy regulation. Although hypoxic conditions can temporarily activate FUNDC1‐mediated autophagy, prolonged hyperglycemia inhibits lysosomal function, impairing the fusion of autophagosomes with lysosomes and negatively affecting the mitophagy process [59]. In diabetes, this inhibition of lysosomal function is likely related to mTORC1 activity. Inhibiting mTORC1 can activate lysosomal function, while increased mTORC1 activity leads to lysosomal dysfunction, hindering autophagosome–lysosome fusion [63]. Furthermore, in a high‐glucose environment, insufficient lysosomal acidification may reduce lysosome numbers, further obstructing autophagosome–lysosome fusion [64].

Chronic inflammation in the diabetic microenvironment also plays a critical role in mitophagy dysfunction. Hyperglycemia and oxidative stress cause mitochondrial damage, triggering the activation of the NLRP3 inflammasome [65]. This activation exacerbates inflammation and impairs mitophagy, preventing the clearance of damaged mitochondria and creating a vicious cycle of increased inflammation and further mitophagy impairment [66]. Moreover, dysregulated mitophagy can lead to the leakage of mitochondrial contents, and the release of DAMPs further activates the NLRP3 inflammasome, promoting the inflammatory response [67].

### 4.2 The Impact of Mitophagy Dysregulation on the Bladder Microenvironment

Mitophagy is a critical mechanism for maintaining mitochondrial function and cellular homeostasis. When mitophagy becomes dysregulated, mitochondrial dysfunction and excessive accumulation of mtROS occur. This accumulation activates various signaling pathways, including pro‐fibrotic and proinflammatory pathways, exacerbating the disruption of the bladder microenvironment [68]. Excessive mtROS production can affect cellular function through multiple pathways. For example, mtROS can activate the nuclear factor‐κB (NF‐κB) signaling pathway, increasing the expression of inflammatory factors and intensifying the inflammatory response [69]. Additionally, mtROS can alter the mitochondrial membrane potential, inducing mitochondrial membrane permeability transition, which further exacerbates cellular damage and inflammation [58]. In the bladder microenvironment, mitophagy defects can lead to ECM remodeling and fibrosis, a process potentially linked to elevated pro‐fibrotic factors like transforming growth factor‐β (TGF‐β), triggered by mtROS [70].

Mitophagy dysregulation can also lead to increased intracellular oxidative stress, which further triggers inflammation and cell death [18]. Studies have shown a strong connection between oxidative stress and mitochondrial damage. Oxidative stress not only damages mtDNA but also aggravates cellular damage by activating inflammatory pathways. For instance, mtDNA damage can activate the cGAS‐STING pathway, triggering an inflammatory response, a process observed in various pathological conditions, including DBD [71]. Additionally, mitophagy defects may disrupt cellular metabolic reprogramming and differentiation, potentially worsening tissue function [72].

Furthermore, mitophagy dysregulation can lead to an imbalance in energy metabolism, exacerbating cellular damage. As the cell's energy source, mitochondria are responsible for ATP synthesis to meet cellular energy demands. The accumulation of dysfunctional mitochondria reduces ATP production, impairing cellular energy supply and weakening the repair function of the autophagy‐lysosome system through the AMPK/mTOR pathway. This creates a vicious cycle of energy crisis and mitophagy defects [18, 73]. This imbalance in energy metabolism leads to insufficient energy for BSMCs, affecting bladder storage and voiding functions [74]. Additionally, mitochondrial dysfunction exacerbates tissue damage by activating apoptosis pathways [75].

### 4.3 Bidirectional Feedback Mechanism

**FIGURE 2:**
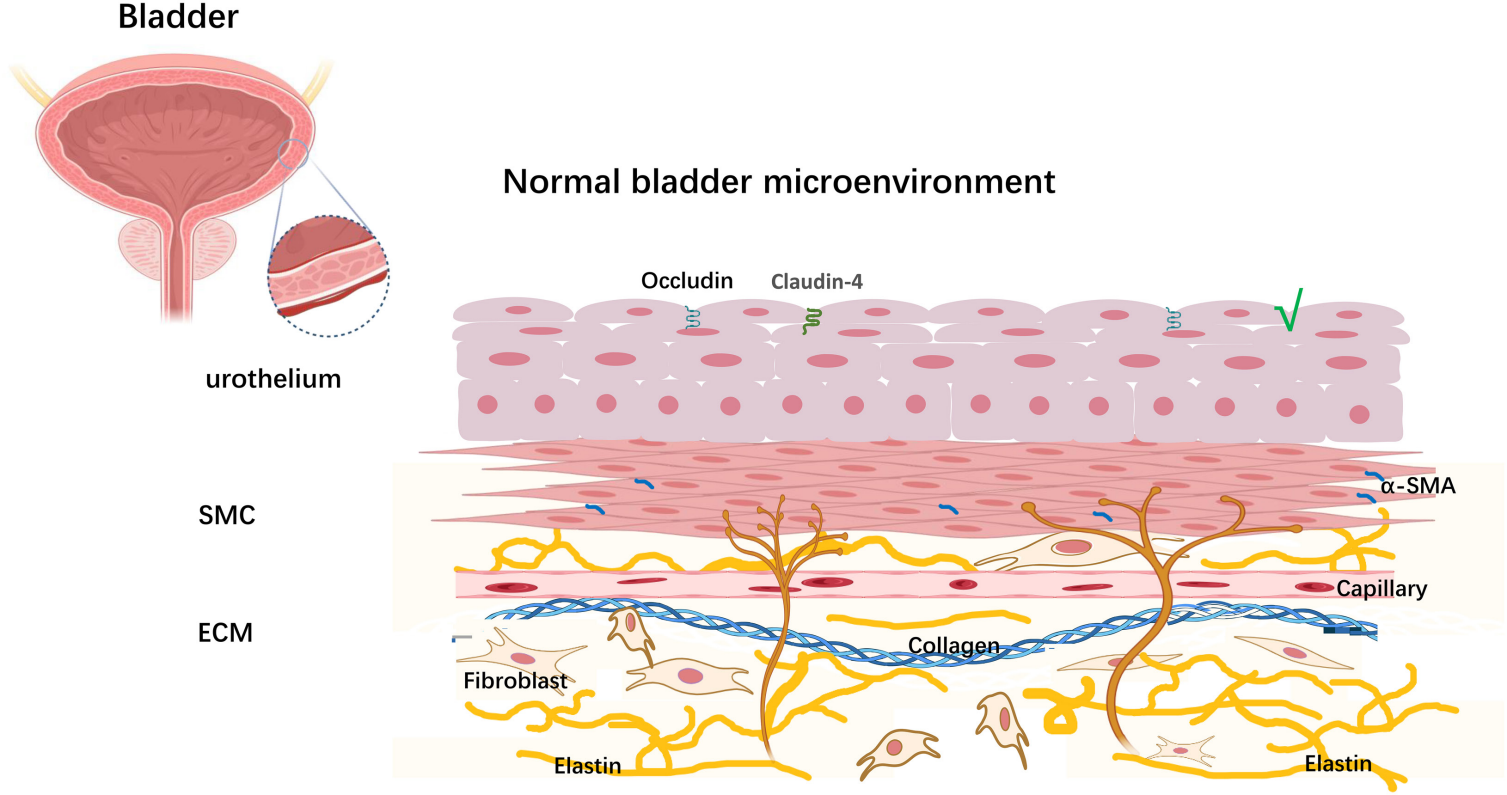
Normal bladder microenvironment: structure and molecular composition. This figure depicts the multilayered structure and molecular composition of a healthy bladder microenvironment, providing a physiological baseline for comparison with the pathological changes shown in Figure 3. The urothelium, a key barrier, is formed by tight junction proteins like Occludin and Claudin‐4, preventing the permeation of urinary toxins. Beneath the urothelium, the SMC, characterized by high α‐SMA expression, enables coordinated contraction and relaxation for efficient voiding. The ECM supports the tissue structure and compliance through a balanced network of collagen and elastin, with fibroblasts maintaining this homeostasis. A well‐organized capillary network ensures the adequate supply of oxygen and nutrients, sustaining tissue viability and neural function. The integrated functions of these components are critical for normal bladder sensation, storage, and emptying. α‐SMA, α‐smooth muscle actin; ECM, extracellular matrix; SMC, smooth muscle cell.

**FIGURE 3:**
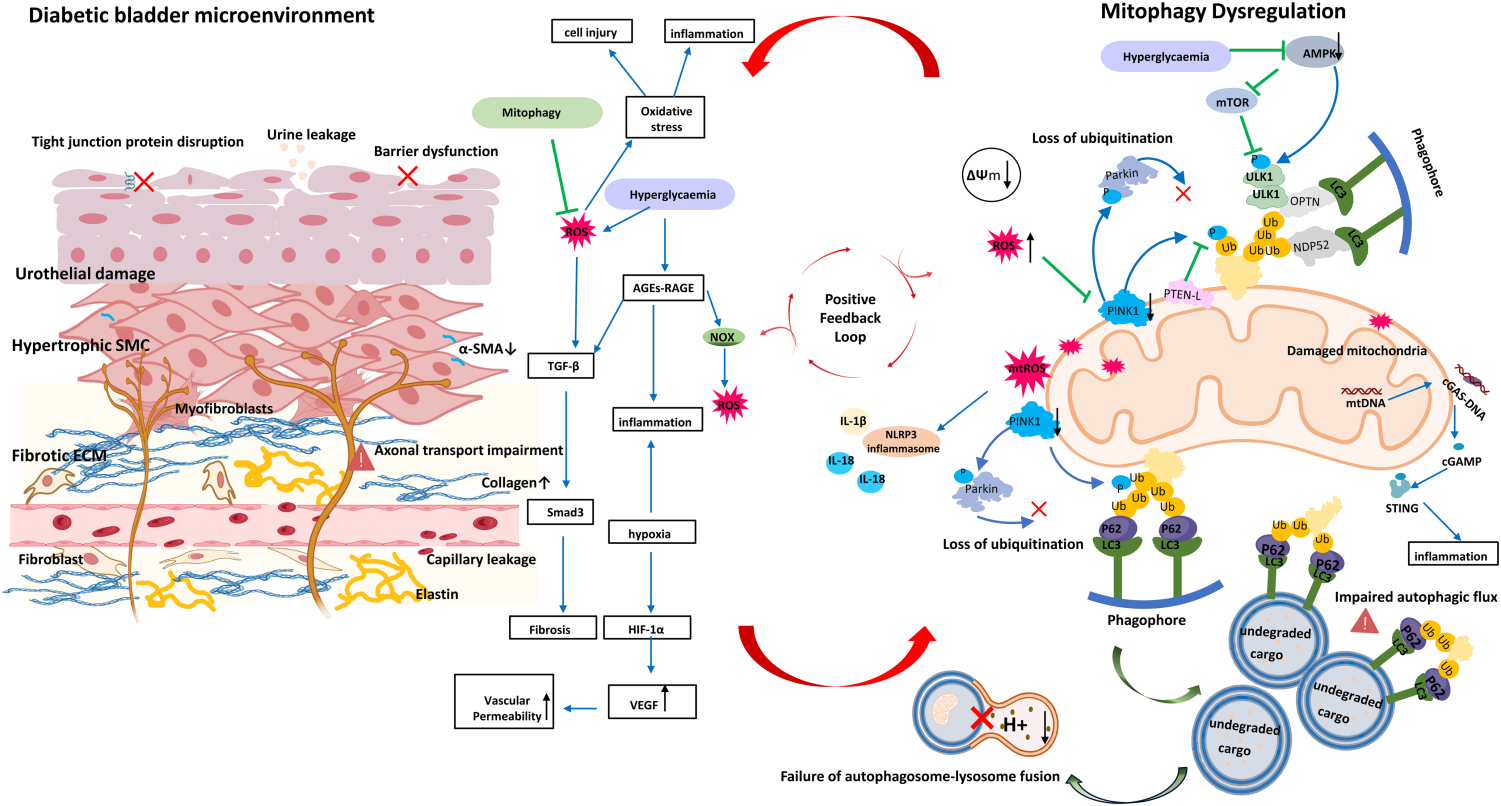
Mitophagy dysregulation and diabetic bladder microenvironment: pathological feedback loop. This figure illustrates the central thesis of our research, showing the vicious cycle that drives DBD progression. The left panel depicts the deteriorating bladder microenvironment under diabetic conditions, where hyperglycemia leads to the generation of AGEs and ROS. These factors damage the urothelial barrier, induce capillary leakage via VEGF upregulation, and trigger chronic inflammation and fibrosis through pathways like TGF‐β. Simultaneously, as shown in the right panel, this hostile microenvironment directly impairs mitophagy via key defects, including suppression of the PINK1/Parkin pathway, inhibition of AMPK signaling, and failure of autophagosome–lysosome fusion. The resulting accumulation of dysfunctional mitochondria generates excessive mtROS and mtDNA. These molecules, acting as DAMPs, activate pro‐inflammatory cascades, including the NLRP3 inflammasome and cGAS‐STING pathway, as well as pro‐fibrotic signaling. Importantly, this exacerbated microenvironmental disruption further suppresses mitophagy, amplifying a self‐sustaining pathological loop. This diagram contrasts the physiological processes in Figure 1 and the intact tissue structure shown in Figure 2. ΔΨm, mitochondrial membrane potential; AGEs, advanced glycation end products; cGAMP, cyclic GMP‐AMP; DAMPs, damage‐associated molecular patterns; ECM, extracellular matrix; HIF‐1α, hypoxia‐inducible factor 1‐α; IL, interleukin; LC3, microtubule‐associated protein 1 light chain 3; mtDNA, mitochondrial DNA; mtROS, mitochondrial reactive oxygen species; NF‐κB, nuclear factor κ‐light‐chain‐enhancer of activated B cells; NOX, NADPH oxidase; P62, sequestosome 1; PINK1, PTEN‐induced kinase 1; RAGE, receptor for advanced glycation end products; ROS, reactive oxygen species; SMC, smooth muscle cell; STING, stimulator of interferon genes; TGF‐β, transforming growth factor‐β; VEGF, vascular endothelial growth factor.

The interplay between mitophagy dysregulation and microenvironmental disruption in DBD culminates in a self‐perpetuating pathological cycle, as schematically depicted in Figure 3. This central concept can be examined by contrasting the pathological state in Figure 3 with the physiological baselines established in Figures 1 and 2. The cycle begins when the diabetic milieu, marked by hyperglycemia and oxidative stress as shown in the left panel of Figure 3, assaults the bladder microenvironment. This damage starkly contrasts with the healthy structure presented in Figure 2. Simultaneously, these insults suppress the core mitophagy machinery. The well‐coordinated PINK1/Parkin and receptor‐mediated pathways outlined in Figure 1 are inhibited, leading to the accumulation of damaged mitochondria, a key feature highlighted in the right panel of Figure 3 under “Mitophagy Dysfunction.” This failure in mitochondrial quality control results in excessive mtROS production and the release of DAMPs, which further activate the NLRP3 inflammasome and pro‐fibrotic signaling, thereby worsening microenvironmental inflammation and fibrosis, as depicted throughout Figure 3. The increasingly hostile microenvironment further represses mitophagy, closing the vicious loop. Figure 3 synthesizes the central thesis: the diabetic bladder microenvironment disrupts mitophagy, which contrasts sharply with the efficient process shown in Figure 1, while the resulting mitophagy failure exacerbates microenvironmental disruption, deviating significantly from the homeostasis depicted in Figure 2. This interaction creates a pathological positive feedback loop that drives DBD progression.

This self‐perpetuating cycle not only clarifies the core pathology of DBD but also identifies critical therapeutic targets, such as restoring mitophagy flux and modulating microenvironmental factors like oxidative stress and fibrosis.

This self‐reinforcing cycle not only clarifies the core pathology of DBD but also establishes a structured network of interdependent therapeutic targets. The cycle's architecture suggests that interventions must extend beyond singular pathways to concurrently address its upstream and downstream components. Effective strategies likely require a dual approach that both restores mitochondrial quality control and mitigates the resultant microenvironmental damage. This imperative is reinforced by evidence that similar mitophagy‐microenvironment feedback loops operate in other diabetic complications, such as nephropathy, where analogous interactions accelerate renal fibrosis and dysfunction [76, 77]. The conservation of this pathogenic motif across tissues underscores its fundamental role in diabetic organ injury and highlights the potential for therapeutic strategies targeting this axis to have broad applicability. In DBD, disrupting this specific vicious cycle is therefore a critical objective for achieving disease modification.

### 4.4 Intercellular and Molecular Communication

The interaction between mitophagy dysregulation and microenvironmental changes involves multiple key signaling pathways. ROS play a central role in regulating mitophagy. Excessive ROS production can suppress the PINK1/Parkin pathway by activating the c‐Jun N‐terminal kinase (JNK) and NF‐κB signaling pathways [78, 79]. Activation of these pathways is associated with cellular stress and inflammation, potentially exacerbating pathological changes in DBD. The PINK1/Parkin pathway is essential for mitochondrial quality control and mitophagy. However, elevated ROS levels may inhibit PINK1/Parkin pathway activity, reducing mitophagy efficiency [80]. The activation of these signaling pathways further aggravates cellular damage, creating a continuous loop of oxidative stress and impaired autophagy.

AMPK serves as a key sensor of cellular energy, activated under low energy conditions to promote autophagy initiation and lysosome formation by inhibiting the mTOR pathway. In diabetes, however, reduced AMPK activity results in overactivation of the mTOR pathway, which suppresses the regular progression of autophagy [81]. In the bladder, this imbalance in energy metabolism can impair urine storage and voiding functions, contributing to urinary dysfunction [82]. Furthermore, decreased AMPK activity exacerbates tissue damage by activating the apoptosis pathway. Inactivation of AMPK impairs cellular responses to energy stress, increasing the risk of cell apoptosis [83]. Thus, the cell is unable to clear damaged organelles and proteins through autophagy, leading to the accumulation of harmful substances and further tissue damage [84].

Additionally, intercellular communication can regulate mitophagy‐related gene expression through miRNAs carried by secreted exosomes. For example, miR‐21 enhances AKT signaling by targeting PTEN, which inhibits Parkin's mitochondrial localization, thereby disrupting the normal mitophagy process [85].

Collectively, the evidence outlines a self‐perpetuating pathological cycle in DBD: hyperglycemia‐induced microenvironmental disruption impairs mitophagy, which exacerbates mitochondrial dysfunction and further deteriorates the microenvironment through increased ROS, fibrosis, and energy crises. This understanding of the core pathological feedback loop raises the critical question: how can we therapeutically intervene to break this cycle? The following section evaluates potential strategies targeting both mitophagy and the microenvironment, individually or in combination.

## 5 Therapeutic Perspectives and Future Directions: Breaking the Cycle

### 5.1 Targeting the Cycle: From Pharmacological Agents to Gene Editing

Given the elucidated vicious cycle between mitophagy dysregulation and microenvironment disruption in DBD, the following sections evaluate therapeutic strategies targeting these interconnected pathways to break the pathological feedback loop. Restoring mitophagy function stands as a core strategy. Pharmacological agents such as urolithin A have shown promise by activating the PINK1/Parkin pathway, enhancing mitochondrial clearance, reducing ROS accumulation, and alleviating bladder fibrosis in diabetic models [86]. Similarly, the antidiabetic drug metformin can improve mitochondrial quality control by activating the AMPK/SIRT1 pathway, thereby facilitating autophagosome–lysosome fusion and restoring mitochondrial respiratory function [86, 87]. Beyond conventional drugs, gene‐editing technologies offer a precise approach. CRISPR/Cas9‐mediated targeting of the Parkin gene has been demonstrated to enhance mitophagy efficiency, leading to improved bladder contraction and reduced fibrosis in diabetic mice [87, 88]. Concurrently, advancements in nanodelivery systems provide a means for targeted therapy. For instance, liposomes encapsulating PINK1 mRNA can be designed to specifically deliver this key mitophagy initiator to bladder tissue, effectively restoring mitophagy flux and improving functional outcomes [74, 89].

Simultaneously, direct modulation of the hostile bladder microenvironment is equally critical. To counteract oxidative stress, antioxidants including *N*‐acetylcysteine can scavenge excess ROS, mitigating oxidative damage to the bladder barrier and nerves [90]. Given the central role of inflammation in DBD progression, targeting the NLRP3 inflammasome with specific inhibitors such as MCC950 has proven effective in blocking IL‐1β and IL‐18 release, thereby reducing bladder inflammation and fibrosis [91, 92]. Furthermore, addressing the fibrotic component of the microenvironment is essential. Modulating pathways involved in ECM remodeling, for example the YAP/Smad3 axis or the Nrf2 pathway using agents like resveratrol, presents a potential strategy to inhibit excessive collagen deposition and tissue stiffening [15, 93].

The interdependence between mitophagy and microenvironmental factors highlights the need for combined therapeutic approaches, as isolated interventions may not effectively disrupt the self‐amplifying pathological cycle. Consequently, a synergistic strategy that concurrently enhances mitophagy using Urolithin A, neutralizes oxidative stress with antioxidants such as N‐acetylcysteine, and suppresses inflammation and fibrosis with agents including MCC950 or resveratrol is hypothesized to be superior to single‐target therapies [86, 90, 93, 94]. The rational design of such combination regimens, potentially aided by nanoparticle co‐delivery systems to ensure coordinated pharmacokinetics, represents a frontier in developing more effective treatments for DBD [95, 96].

### 5.2 Overcoming Current Challenges and Future Avenues

Despite promising therapeutic avenues, significant gaps in knowledge and technology must be bridged to enable clinical translation. A key mechanistic gap lies in understanding the spatiotemporal regulation and pathological contribution of non‐ubiquitin‐dependent mitophagy pathways, including FUNDC1, within the diabetic bladder [97, 98]. Furthermore, the synergistic dysfunction between mitophagy and lysosomal degradation in the diabetic milieu, particularly regarding impaired autophagosome–lysosome fusion, requires further elucidation [59, 99].

To address these complexities, future research must leverage advanced technologies. Spatial transcriptomics and single‐cell sequencing are indispensable tools for mapping the cellular heterogeneity of mitophagy‐related gene expression across diverse bladder cell types including urothelial cells, SMCs, and fibroblasts. This will identify the most vulnerable cellular targets for precise intervention [100, 101]. In parallel, the development of more physiologically relevant in vitro models, such as three‐dimensional bioprinted bladder organoids incorporating vascular and neural networks, is crucial for high‐fidelity mechanistic studies and high‐throughput screening of combination therapies [102].

On the technological front, the next generation of nanodelivery systems must evolve toward greater intelligence and specificity. Designing nanoparticles that can respond to the unique biochemical cues of the diabetic bladder microenvironment, for example high ROS levels or low pH, for triggered drug release will maximize on‐target efficacy while minimizing systemic side effects [103, 104]. The integration of these disruptive technologies—advanced omics for target discovery, sophisticated organoids for validation, and smart nanocarriers for delivery—with a deep understanding of the mitophagy‐microenvironment axis paves the way for mechanism‐driven, personalized combination therapies. This convergent approach holds the ultimate promise of moving beyond symptomatic management to definitively break the vicious cycle at the heart of DBD (Tables 1, 2, 3).

**TABLE 1:** Comparison of the role of mitophagy‐related molecules in diabetic complications.

Molecule/pathway	DBD	Diabetic nephropathy (DN)	Diabetic retinopathy (DR)
PINK1/Parkin	Significant reduction in PINK1/Parkin activity leads to decreased mitochondrial clearance efficiency, exacerbating oxidative stress and fibrosis in the bladder microenvironment [12, 58].	Downregulation of expression in renal tubular epithelial cells and mitophagy defects lead to increased cell apoptosis and interstitial fibrosis [12].	Mitophagy inhibition in retinal ganglion cells exacerbates neurodegeneration [105, 106].
AMPK/mTOR	AMPK activity is inhibited, and excessive activation of mTOR suppresses mitophagy, exacerbating oxidative stress and cellular damage in the bladder microenvironment [107].	Activation of AMPK improves podocyte mitochondrial function, inhibits mTOR, and indirectly alleviates cell damage [108].	Imbalance of AMPK/mTOR disrupts mitochondrial network homeostasis and exacerbates retinal oxidative damage [99].
NLRP3 inflammasome	Mitophagy dysregulation leads to excessive release of mtROS, which induces NLRP3 activation and drives bladder inflammation and fibrosis [43].	Mitochondrial DNA leakage activates NLRP3, promoting tubular inflammation and fibrosis [109].	Retinal vascular leakage is associated with NLRP3‐mediated inflammation [109].

**TABLE 2:** Summary of key research findings on the interaction between mitophagy dysregulation and the diabetic bladder microenvironment.

Research topic	Major findings	Involved molecules/pathways	References
Molecular mechanisms of impaired mitophagy function	In the diabetic bladder microenvironment, the activity of the important PINK1/Parkin pathway in the ubiquitin‐dependent mitophagy pathway significantly decreases, leading to impaired clearance of damaged mitochondria, accumulation of ROS, and exacerbated fibrosis.	PINK1/Parkin, ROS, NLRP3 inflammasome	[12, 17, 58]
Interaction between hypoxia and mitophagy	Chronic hypoxia briefly induces FUNDC1‐mediated mitophagy and activates the HIF‐1α/VEGF axis, exacerbating bladder microenvironment disruption.	FUNDC1, HIF‐1α, VEGF	[33, 49, 59]
The vicious cycle of inflammation and autophagy	High‐glucose‐induced mitochondrial ROS leakage activates NLRP3 inflammasome, releasing IL‐1β/IL‐18, which further inhibits autophagy and drives fibrosis.	NLRP3, IL‐1β, TGF‐β/Smad3	[43, 58, 110]
The impact of AMPK/mTOR pathway imbalance	High glucose inhibits AMPK activity, leading to excessive mTOR activation, obstructing autophagic flux and exacerbating mitochondrial fragmentation.	AMPK/mTOR, ULK1	[81, 107, 111]

**TABLE 3:** Potential therapies targeting mitophagy and microenvironment regulation.

Therapeutic strategy	Mechanism of action	Targeted molecules/pathways	
Urolithin A	Activates the PINK1/Parkin pathway, enhances mitophagy, and reduces ROS accumulation and fibrosis.	PINK1/Parkin, PGC‐1α	[86]
Metformin	Activates the AMPK/SIRT1 pathway, promotes autophagosome–lysosome fusion, and improves mitochondrial respiratory chain function.	AMPK/mTOR, ULK1	[87, 112]
MCC950	Inhibits NLRP3 inflammasome activity, blocks IL‐1β/IL‐18 release, and alleviates inflammation and fibrosis.	IL‐1β/IL‐18, NLRP3 inflammasome	[91]
*N*‐acetylcysteine (NAC)	Scavenges ROS, alleviates oxidative stress damage to bladder barrier and neural function.	ROS	[90]
Resveratrol	Activates the Nrf2 pathway, regulates ECM metabolism, and inhibits fibrosis.	Nrf2, TGF‐β/Smad3	[93]
Combination therapy	NAC (antioxidant) + resveratrol (antifibrotic) + urolithin A (autophagy activation) synergistic treatment.	PINK1/Parkin, ROS， PGC‐1α, Nrf2, TGF‐β/Smad3	[86, 90, 93]
Targeted nanodelivery system	Liposomes encapsulating PINK1 mRNA or miRNA, precisely delivering to bladder tissue, restoring mitophagy function.	PINK1	[74]
CRISPR/Cas9 editing of Parkin	Gene editing restores Parkin expression, specifically enhancing mitophagy efficiency.	Parkin	[88, 113]

## Funding

This work was supported by the National Natural Science Foundation of China, 82370781, 82470808.

## Conflicts of Interest

The authors declare no conflicts of interest.
